# After the first wave and beyond lockdown: long-lasting changes in emergency department visit number, characteristics, diagnoses, and hospital admissions

**DOI:** 10.1007/s11739-021-02667-2

**Published:** 2021-03-08

**Authors:** Fulvio Morello, Paolo Bima, Enrico Ferreri, Michela Chiarlo, Paolo Balzaretti, Gloria Tirabassi, Paolo Petitti, Franco Aprà, Domenico Vallino, Giorgio Carbone, Emanuele Emilio Pivetta, Enrico Lupia

**Affiliations:** 1 S.C. Medicina d’Urgenza U, Ospedale Molinette, A.O.U. Città della Salute e della Scienza and Università degli Studi di Torino, C.so Bramante 88, 10126 Torino, Italy; 2 Dipartimento di Scienze Mediche, Università degli Studi di Torino, Torino, Italy; 3 Scuola di Specializzazione in Medicina d’Emergenza-Urgenza, Università degli Studi di Torino, Torino, Italy; 4 Medicina e Chirurgia d’Accettazione e d’Urgenza, Ospedale Maria Vittoria, Torino, Italy; 5 Medicina e Chirurgia d’Accettazione e d’Urgenza, Ospedale San Giovanni Bosco, Torino, Italy; 6 S.C. Medicina e Chirurgia d’Accettazione e d’Urgenza, A.O. Ordine Mauriziano, Torino, Italy; 7 S.C. Medicina e Chirurgia d’Accettazione e d’Urgenza, Humanitas, Torino, Italy; 8 Scuola di Specializzazione in Medicina d’Emergenza-Urgenza, Humanitas University, Pieve Emanuele, Milano, Italy

**Keywords:** Emergency, Visits, Hospital admission, COVID-19, Coronavirus

## Abstract

The first wave (FW) of COVID-19 led to a rapid reduction in total emergency department (ED) visits and hospital admissions for other diseases. Whether this represented a transient “lockdown and fear” phenomenon, or a more persisting trend, is unknown. We divided acute from post-wave changes in ED flows, diagnoses, and hospital admissions, in an Italian city experiencing a FW peak followed by nadir. This multicenter, retrospective, cross-sectional study involved five general EDs of a large Italian city (January–August 2020). Percent changes were calculated versus 2019, using four 14-day periods (FW peak, early/mid/late post-wave). ED visits were 147,446 in 2020, versus 214,868 in 2019. During the FW peak, visits were reduced by 66.4% (*P* < 0.001). The drop was maximum during daytime (69.8%) and for pediatric patients (89.4%). Critical triage codes were unchanged. Reductions were found for all non-COVID-19 diagnoses. Non-COVID-19 hospital admissions were reduced by 39.5% (*P* < 0.001), involving all conditions except hematologic, metabolic/endocrine, respiratory diseases, and traumas. In the early, mid, and late post-wave periods, visits were reduced by 25.4%, 25.3% and 23.5% (all *P* < 0.001) respectively. In the late period, reduction was greater for female (27.9%) and pediatric patients (44.6%). Most critical triage codes were unchanged. Oncological, metabolic/endocrine, and hematological diagnoses were unchanged, while other diagnoses had persistent reductions. Non-COVID-19 hospital admissions were reduced by 12.8% (*P* = 0.001), 6.3% (*P* = 0.1) and 12.2% (*P* = 0.001), respectively. Reductions in ED flows, led by non-critical codes, persisted throughout the summer nadir of COVID-19. Hospital admissions for non-COVID-19 diseases had transient changes.

**Supplementary Information:**

The online version contains supplementary material available at 10.1007/s11739-021-02667-2.

## Introduction

The first diffusion wave of COVID-19 in early 2020 led to a steep decline in total Emergency Department (ED) visits, contradicting previous experience with seasonal influenza and ED overcrowding [1–4]. This unpredicted phenomenon involved acute drops in the diagnoses and hospital admissions for time-dependent conditions, raising major clinical concerns [5–8]. However, cross-sectional details regarding ED visit characteristics, diagnostic outcomes, and disposed hospital admissions, have been sparse, and reports have focused on the initial phase of the COVID-19 pandemic, when the first lockdown measures were disposed. In several countries, especially in Europe, an epidemiological post-wave nadir phase devoid of lockdown measures was registered during the summer of 2020. Trends in ED flows and activity after the first wave, beyond the initial “public fear and lockdown” response, are unknown. Understanding the dynamics and kinetics of this phenomenon is instrumental to guide organization of EDs, medical wards and hospital care at large, during and beyond the second wave of COVID-19.

In the city of Torino, a large northern Italian city (870,000 inhabitants), during the first wave of the pandemic, the number of daily COVID-19 diagnoses peaked in March–April 2020. Since June, a persistently low incidence was registered throughout the summer of 2020, when restrictive measures were withdrawn. We took advantage of this epidemiological scenario, characterized by a well-defined first wave and post-wave nadir phase, to evaluate ED flows, visit characteristics, diagnoses, and hospital admissions, during the first wave and in the post-wave phase. Our working hypothesis was that these variables would return to previous standards after the resolution of the first wave and withdrawal of lockdown measures. Persistent changes, instead, would prompt to structural and long-lasting effects of the pandemics on use of/referral to EDs.

## Methods

### Study design and setting

We conducted a multicenter, observational, retrospective study in five hospitals in the urban area of Torino, Italy, covering all medical and surgical specialties. Participating centers (ESM Appendix Table 1) were: one large tertiary university hospital (coordinating center), one tertiary non-university hospital, two secondary non-university hospitals and one smaller community hospital. All are public hospitals (except for the latter, which is private providing public healthcare). The overall census of the participating EDs is about 320.000 ED visits/year (pre-COVID-19 period), corresponding to ≈ 90% of ED visits in the urban area. Urban hospitals not participating to the present study were one small community hospital, a specialty trauma center, a specialty obstetrical/gynecological center and a specialty pediatric center.

### Epidemiological scenario and lockdown timing

During the first wave of the pandemic, the national peak of COVID-19 cases was reached on 20–28th March 2020. To reduce viral diffusion, a national lockdown was imposed from 9th March to 17th May 2020. On 3rd June 2020, key restrictive measures were withdrawn, and from May, no excess mortality was registered across the nation [9]. In the city of Torino, the number of daily COVID-19 diagnoses peaked at about 300/day from 20th March to 14th of April 2020, and stably returned at < 10/day from 18th of June, with low incidence persisting throughout the summer.

### Measurements

The study period was 1st January to 31st August, 2020 and 2019. ED data were extracted in each center from the health record database in anonymized form. An automatic query was performed to extract the following data from each visit: patient gender, age, date of ED registration, triage priority, time of arrival, triage main symptom, date of discharge, patient destination (discharge *vs* hospital admission), and final diagnosis. Data extraction did not involve evaluation of individual medical charts or registration of additional personal/clinical information, and data treatment conformed to Italian D. Lgs. 196/2003 and European regulation 2016/679. Since the study focused on general ED flow analysis (system-level) and not on individual patients, only utilized anonymized data, and was retrospective observational in nature, Ethic Committee approval was waived, as confirmed by the Hospital Board (prot. 101035, 23rd October 2020).

### Triage codes and symptoms

Triage priority was based on standard triage nurse evaluation using color-codes following national indications. Briefly, a red code is attributed when a patient is suspected of having a life-threatening condition or vital sign modification, implying immediate evaluation. A yellow code is given when a patient displays signs/symptoms that could underlie a serious illness with evolving risk, implying medical visit within 30 min. A green code is assigned when a patient does not present warning signs/symptoms, and whose medical evaluation can be deferred. A white code is attributed when a patient complains symptoms that could be evaluated during an ambulatory medical visit in the following hours.

Triage evaluation includes a structured interview by a trained nurse, also defining main triage symptom. From March 2020, systematic triage assessment of key COVID-19 symptoms was also applied, to allow early allocation of patients satisfying criteria to a dedicated ED area. Color code evaluation was unchanged. In data analysis, the following main triage symptoms were queried: dyspnea, chest pain, abdominal pain, psychiatric symptom.

### Diagnostic classification

Discharge diagnoses were grouped using the ICD-9-CM classification, with few modifications based on clinical reasoning. Within the ICD group “16–symptoms, signs, and ill-defined conditions”, selected diagnoses were analyzed per se because frequently leading to ED visits (syncope, unspecific chest pain), or were grouped within organ-specific categories (ESM Appendix Table 2). Fever and sepsis/septic shock were grouped within infectious diseases, dyspnea was grouped within respiratory diseases, palpitations were grouped within cardiovascular diseases, and convulsions were grouped within neurological diseases. Pediatric patients were defined by age < 14 years. Based on the local practice of all participating centers, ICD-9-CM codes defining SARS-CoV-2 infection were: 79.82, 480.3 or V01.82.

### Statistical analysis

For statistical analysis, the study focused on four 14-day periods in 2020, which were compared to the corresponding periods in 2019: 31st March–13th April (climax of the first wave), 16th–29th June (early post-wave), 14th–27th July (mid post-wave) and 18th–31st August (late post-wave period). The last three study periods were chosen a priori to be evenly distributed, allowing two weeks of adaptation after withdrawal of lockdown measures.

Count data were expressed with absolute number and proportion. Using the Poisson regression, we estimated the percent change and its 95% confidence interval (CI) from the exponentiated Poisson regression coefficient. Type-III *P* values were used to assess whether the Poisson regression model with a specific variable was statistically significant (*P* value < 0.05). Data were displayed using locally estimated scatterplot smoothing (LOESS), in order better show data trend (smoothing span conservatively set at 14%). Extraction of count data and graphs were done with Microsoft Excel (Microsoft Corp., ver. 16.0), MedCalc (MedCalc Software Ltd, ver. 19.5.2), and all statistical analyses were performed using SPSS (IBM Corp., ver. 25.0).

## Results

### Visit number and characteristics

**Fig. 1:**
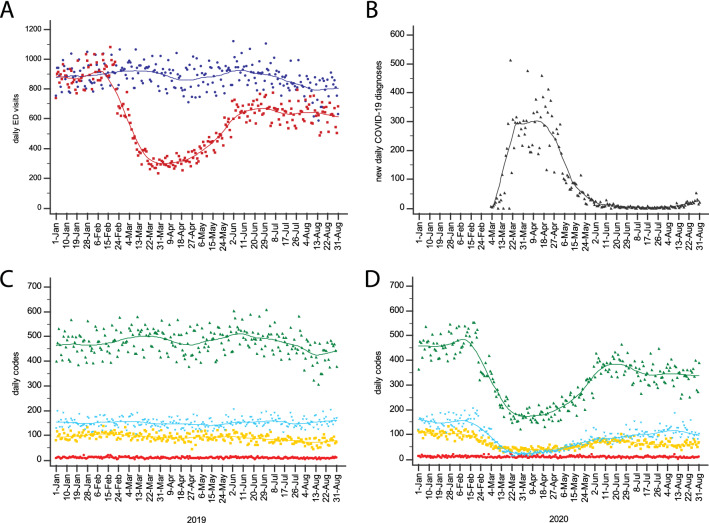
**a** Daily number of ED visits in 2020 (red) and 2019 (blue), from 1st January to 31st August. **b** Number of new daily diagnoses of COVID-19 in the Torino province, in 2020. **c** Daily number of red (most urgent/critical; red dots/line), yellow (urgent for evolutive risk; yellow dots/line) green (non-urgent, deferrable; green dots/line) and white codes (least urgent, deferrable to ambulatory clinic; light blue), in 2019. **d** Daily number of red, yellow, green and white codes, in 2020 (Color figure online)

In the study centers, total ED visits were 147,446 from January to August 2020, and 214,868 in the corresponding period of 2019. The number of daily ED visits is shown in Fig. 1a. Trends were similar across all centers (ESM Appendix Fig. 1). The incidence of COVID-19 diagnoses identified 1st March to 15th May 2020 as the first wave period (Fig. 1b).

During the first wave peak, ED visits were reduced by 66.4% compared to 2019 (*P* < 0.001; Table 1). The reduction was 69.8% during daytime (8 a.m. to 4 p.m.; *P* < 0.001), 64.1% in the evening (4 p.m. to 0 a.m.; *P* < 0.001) and 58.6% during nighttime (*P* < 0.001). The reduction in ED visits was 71.7% (*P* < 0.001) for patients aged < 50 years and 61.7% for patients aged > 75 years (*P* < 0.001). A maximum 89.4% reduction was found for pediatric patients (*P* < 0.001). The first wave peak reduction in ED visits was similar for female and male patients (64.4% and 68.4%; both *P* < 0.001).

**Table 1:** ED visit count and corresponding % change in the selected periods

Period analyzed	2019	2020	% change (95% CI)	*P* value
	ED visit count	ED visit count		
First-wave peak (31st Mar–13th Apr)	12,420	4168	66.4% (65.2–67.6)	< 0.001
Early post-wave (16th–29th Jun)	12,609	9404	25.4% (23.4–27.4)	< 0.001
Mid post-wave (14th–28th Jul)	11,768	8796	25.3% (23.2–27.3)	< 0.001
Late post-wave (18th–31st Aug)	11,365	8693	23.5% (21.3–25.6)	< 0.001

The reduction in total ED visits persisted also after the first COVID-19 wave, by 25.4%, 25.3% and 23.5% (all *P* < 0.001) in the early, mid and late post-wave periods, respectively. In the post-wave periods, reductions were homogenous across daytime and adult patient age (ESM Appendix Table 3). ED visits for pediatric patients were substantially and persistently reduced in all post-wave periods, by 57.6%, 39.9% and 44.6% (all *P* < 0.001) in the early, mid and late post-wave periods, respectively. Reductions in ED visits were persistently larger for female than for male patients: 33.6% vs 17.5% in the early, 29.3% vs 22.2% in the mid, 27.9% vs 19.1% in the late post-wave period (all *P* < 0.001).

### Triage data

Within triage priority, most urgent/critical codes were unchanged in 2020 compared to 2019, both during and after the first wave (Fig. 1c). Significant reductions were observed for less urgent codes, both within the first wave peak and in the post-wave periods (Fig. 1d). The greatest reductions were observed for most deferrable cases (ESM Appendix Table 4). In the late post-wave period, yellow, green and white codes were persistently reduced by 19.4%, 20.2% and 34.7% (all *P* < 0.001), respectively.

All main triage symptoms showed a statistically significant reduction, both during and after the first wave (ESM Appendix Table 4). The lowest change was registered for dyspnea, which presented similar reductions during (13.8%; *P* = 0.011) and after (19.8%, *P* = 0.001; 15.9%, *P* = 0.016; 14.8%, *P* = 0.017) the first wave.

### ED diagnoses

**Fig. 2:**
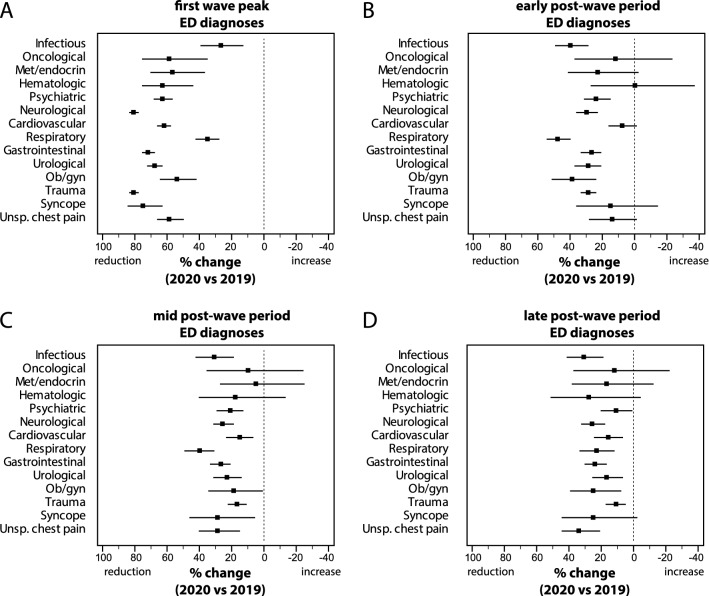
Percent change (with 95% CI) in ED diagnoses other than COVID-19, during **a** the first wave peak (31st March–13th April 2020), **b** early post-wave period (16th–29th June 2020, **c** mid post-wave period (14th–27th July 2020), and **d** late post-wave period (18th–31st August 2020), compared to the corresponding periods of 2019

During the first wave peak, all non-COVID-19 diagnoses showed a significant reduction compared to 2019 (Fig. 2 and ESM Appendix Table 5), ranging from a minimum of 27.2% (*P* < 0.001) for infectious diseases to a maximum of 80.8% (*P* < 0.001) for neurologic diseases. After the first wave, diagnoses of oncological, metabolic/endocrine and hematological diseases were statistically unchanged, in the early, mid and late periods. Infectious, psychiatric, neurological, non-COVID-19 respiratory, gastrointestinal, urological, obstetrical/gynecological diseases and trauma showed a significant reduction in all post-wave periods. In the late period, reductions ranged from a minimum of 10.7% (*P* = 0.034) for psychiatric diseases to a maximum of 30.8% (*P* < 0.001) for infectious diseases. Cardiovascular diseases and unspecific chest pain were statistically unchanged in the early post-wave period and reduced subsequently (15.8% and 33.7% respectively in the late period, both *P* < 0.001).

### Hospital admissions

**Fig. 3:**
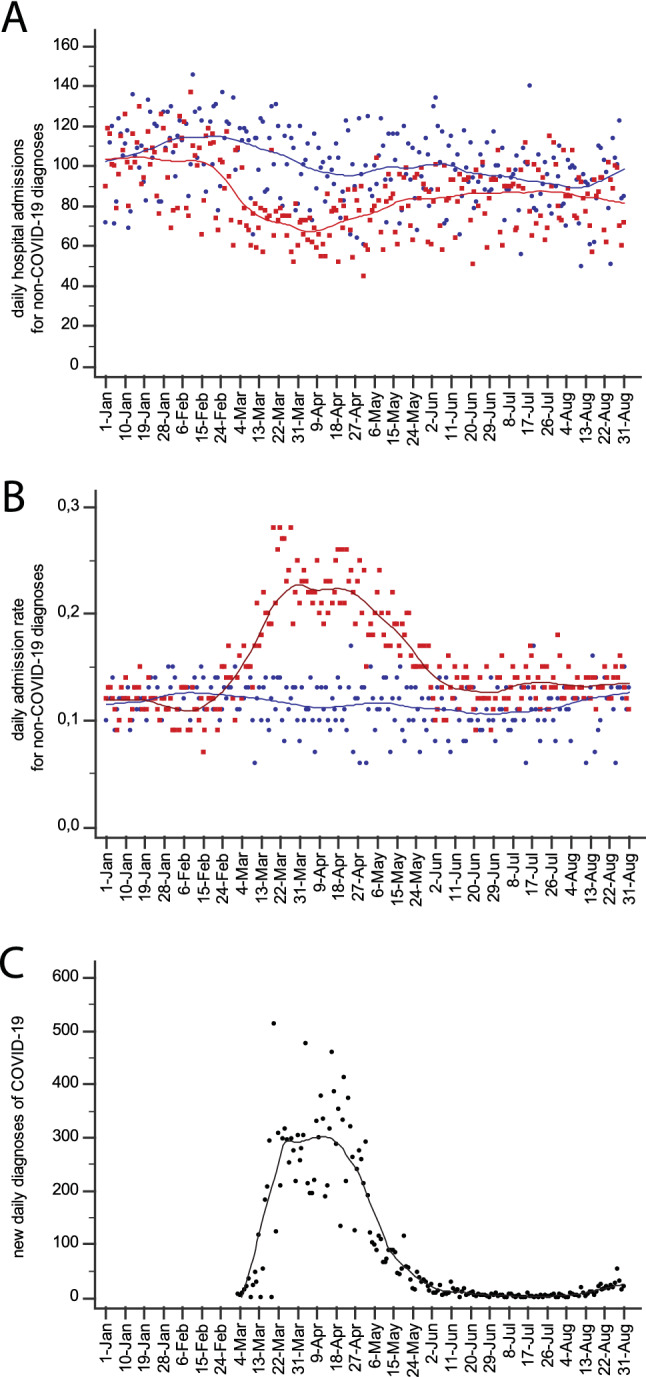
**a** Daily hospital admissions and **b** hospitalization rate from 1st January to 31st August 2020 (red dots/line) and 2019 (blue dots/line). **c** Daily number of new COVID-19 diagnoses in the Torino province in the same period of 2020 (Color figure online)

Total ED-disposed hospital admissions for non-COVID-19 diseases are shown in Fig. 3a. Non-COVID-19 admissions were reduced by 39.5% (*P* < 0.001) during the first wave peak, and by 12.8% (*P* = 0.001), 6.3% (*P* = 0.104) and 12.2% (*P* = 0.001) in the early, mid and late post-wave period, respectively (ESM Appendix Table 6). The admission rate (Fig. 3b) reached a climax during the first wave, with a slight but persistent increase throughout the post-wave period (in the late period, 13.5% *vs* 11.9% in 2019).

**Fig. 4:**
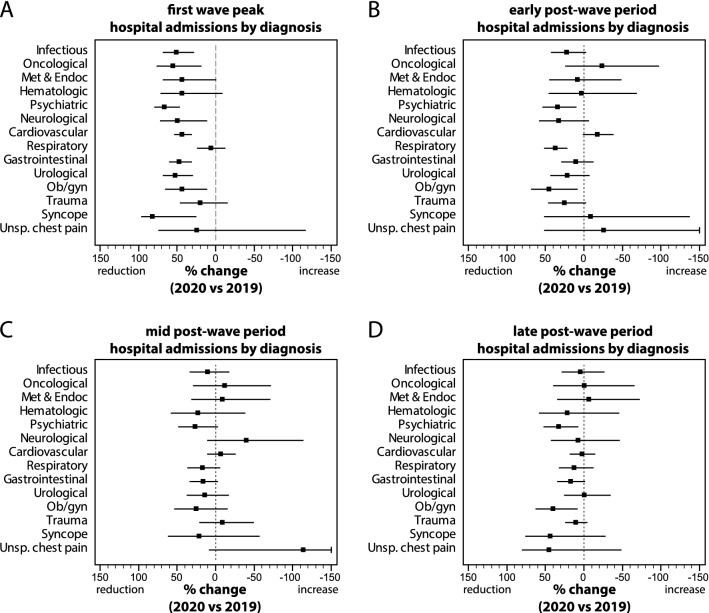
Percent change (with 95% CI) in hospital admissions for non-COVID-19 diseases, during **a** the first wave peak (31st Mar–13th April 2020), **b** early post-wave period (16th–29th June 2020), **c** mid post-wave period (14th–27th July 2020), and **d** late post-wave period (18th–31st August 2020), compared to the corresponding periods of 2019

Percent changes in hospital admissions for non-COVID-19 diagnoses are shown in Fig. 4 and detailed in ESM Appendix Table 6. During the first wave peak, admissions were significantly reduced for infectious, oncological, psychiatric, neurological, cardiovascular, gastrointestinal, urological, obstetric/gynecological diseases and syncope (Fig. 4a), ranging from a minimum of 43.6% (*P* < 0.001) for cardiovascular diseases to a maximum of 83.3% (*P* = 0.019) for syncope. In the post-wave period, hospital admissions were significantly reduced in the early period for psychiatric, non-COVID-19 respiratory and obstetric/gynecological diseases (Fig. 4b), and in the late period for psychiatric and obstetric/gynecological diseases (Fig. 4d).

## Discussion

This is the first study analyzing the long-term effects of COVID-19 on ED flows beyond the early diffusion phase and throughout the epidemiological nadir observed during the summer of 2020 in European countries [2, 4, 5]. Reductions, during and after the first wave peak, mostly derived from non-urgent codes, i.e. cases amenable to deferrable evaluation and potentially indicative of ED misuse. Instead, the number of urgent codes was unchanged. During the wave peak, changes were more pronounced during daytime and in younger patients, also indicating a prevalent effect on deferrable cases. The drop was very substantial and persistent for pediatric patients, as previously reported [10, 11]. In the post-wave periods, the reduction in ED visits was also more pronounced for female patients, indicating gender-specific differential effects.

In line with previous reports, we also found a major reduction in cardiovascular and neurologic diagnoses during the wave peak, but moderate reductions also persisted in the post-wave phase [5–8]. Related hospital admissions normalized after the peak, potentially indicating a prevalent reduction in milder cases. For several other conditions, the post-wave period was also characterized by a long-lasting reduction in ED diagnoses, while admissions were normalized. Overall, these data indicate potential ED undertreatment as a COVID-19 side effect, but also that several patients may have been managed outside EDs [12]. Both ED diagnoses and admissions for onco-hematological and metabolic/endocrine diseases returned to pre-COVID-19 standards early after the first wave, potentially indicating lower disposal or efficiency of alternative care to EDs for these conditions.

Admission data indicate that, in spite of a long-lasting decline in ED visits, the number of hospital beds needed for non-COVID-19 diseases after a wave is largely unchanged, especially for patients affected by metabolic/endocrine and hematologic diseases. Results also show that during a pandemic peak, the admission rate for non-COVID-19 conditions reaches a climax, likely due to burn-out of ED resources by COVID-19, increased difficulties in outpatient management, loss of beds in ED-driven observation units, and increased proportion of patients with more severe conditions seeking ED care.

While facing a second COVID-19 wave worldwide, study results have practical implications. Reduced numbers of patients accessing EDs constitutes an unprecedented opportunity to preserve or even increase the quality of ED care. However, additional barriers may counterbalance the potential benefits of visit reduction, such as the necessity of pre-triage, additional testing, and patient distancing. Unless well designed strategies are put in place, insufficient ED facilities and long turnaround times for testing will prevail, leading to dangerous overcrowding and increased boarding time.

The current study does not provide data on the causes leading to ED visit reduction, and quantification of patients inappropriately avoiding or delaying ED visits was not possible. Differential effect on women, pediatric patients and triage codes indicate that this is likely a multifactorial phenomenon, warranting further category-specific investigation.

The study has limitations. First, results essentially apply to areas experiencing a clear-cut viral wave followed by nadir. Second, sub-analyses of ED discharge diagnosis must be interpreted with caution. Such diagnosis, chosen by the attending physician as the prevalent diagnosis, may not correspond to subsequent medical evaluations and may represent the prevalent, but not the only, clinically meaningful condition. Third, sub-analyses are less powered for infrequent disease categories (thus leading to type II error). Fourth, visits for most severe obstetrical/gynecological diagnoses, traumas and pediatric patients may be under-represented (selection bias), because locally, these cases are frequently conducted to the corresponding specialty ED not included in the present study. In general, reductions in ED visits, diagnoses and hospital admissions may be less pronounced in referral centers accepting most severe cases from large areas.

In conclusion, we found that the first wave of COVID-19 modified ED flows in the long-term, with measurable changes during the summer of 2020, after withdrawal of lockdown measures and return of local COVID-19 incidence to a minimum. Reductions in total visits were dragged by non-urgent triage codes, and more pronounced for female and pediatric patients. Hospital admissions acutely declined during the first wave, but rapidly returned post-peak almost to previous standards. Admissions for certain conditions (metabolic/endocrine and hematologic diseases), however, were unchanged even during the peak, indicating that a sufficient number of hospital beds for non-COVID-19 diseases must be guaranteed throughout a peak, rapidly reverting to standard numbers after the peak resolution. Finally, this unprecedented reduction in ED visits should be regarded as a proof-of-concept that ED overuse and overcrowding are hard yet affordable endpoints for strong healthcare policies.

## Supplementary Information

Below is the link to the electronic supplementary material.

Supplementary file1 (PDF 1238 KB)

## Data Availability

Not applicable.
