# Supplementary material for: After the first wave and beyond lockdown: long-lasting changes in emergency department visit number, characteristics, diagnoses, and hospital admissions
Source: Intern Emerg Med. 2021 Mar 8;16(6):1683–90. doi: 10.1007/s11739-021-02667-2 (PMC7938273; doi:10.1007/s11739-021-02667-2)
Supplement: Supplementary file 1 — Supplementary file1 (PDF 1238 KB) [file 11739_2021_2667_MOESM1_ESM.pdf]

After the first wave and beyond lockdown: long-lasting changes in emergency department visit number, characteristics, diagnoses, and hospital admissions

Fulvio Morello M.D. Ph.D., Paolo Bima M.D., Enrico Ferreri M.D., Michela Chiarlo M.D., Paolo Balzaretti M.D., Gloria Tirabassi M.D., Paolo Petitti M.D., Franco Aprà M.D., Domenico Vallino M.D., Giorgio Carbone M.D., Emanuele Emilio Pivetta M.D., Enrico Lupia M.D. Ph.D.

APPENDIX SUPPLEMENTARY DATA

Appendix Table 1. Characteristics of participant hospitals.

Hospital name	2019 ED visits	2019 hospital admissions	2019 mean admission rate	Academic site	Hospital type
Maria Vittoria	84000	8100	9.6%	No	Secondary
Molinette	70500	14500	20.6%	Yes	Large tertiary referral
San Giovanni Bosco	69000	5550	8.0%	No	Tertiary referral
Ordine Mauriziano	57000	6200	10.9%	No	Secondary
Humanitas Gradenigo	41000	2200	5.4%	No	Community

Appendix Table 2. ICD-9-CM grouping codes.

Pathological group	ICD-9-CM codes
Infectious diseases	001-139; 785.52; 780.6; 995.90-995.99
Oncological diseases	140-239
Metabolic and endocrine diseases	240-279
Hematological diseases	280-289
Psychiatric diseases	290-319
Neurological diseases	320-389; 780.3; 784.0-784.99
Cardiovascular diseases	390-459; 785.0-785.51; 441.0-441.99; 997.1; 997.91
Respiratory diseases	460-519; 786.0-786.49
Gastrointestinal diseases	520-579; 787.0-787.99; 789.0-789.99
Urological diseases	580-629; 788.0-788.99
Obstetrical and gynecological diseases	610-611.9; 614-677
Trauma and burns	800-959.9
Syncope	780.2
Unspecific chest pain	786.50-786.59

Appendix Table 3. ED visit count classified by patient gender, age and time of arrival in the selected periods, with corresponding % change.

	31st Mar-13th Apr (peak period)				16th Jun-29th Jun (early post-peak period)				14th Jul-27th Jul (mid post-peak period)				18th Aug-31st Aug (late post-peak period)			
	2020	2019			2020	2019			2020	2019			2020	2019		
	cum. No.	cum. No.	%ch.	*P*	cum. No.	cum. No.	%ch.	*P*	cum. No.	cum. No.	%ch.	*P*	cum. No.	cum. No.	%ch.	*P*
**General characteristics**																
Female sex	1967 (47.2%)	6234 (50.2%)	68.4% (66.8-70.0)	<0.001	4128 (43.9%)	6217 (49.3%)	33.6% (30.9-36.2)	<0.001	4140 (47.1%)	5856 (49.8%)	29.3% (26.4-32.1)	<0.001	4111 (47.3%)	5701 (50.2%)	27.9% (24.9-30.7)	<0.001
Male sex	2201 (52.8%)	6186 (49.8%)	64.4% (62.6-66.1)	<0.001	5276 (56.1%)	6392 (50.7%)	17.5% (14.4-20.4)	<0.001	4656 (52.9%)	5912 (50.2%)	22.2% (18.2-24.2)	<0.001	4582 (52.7%)	5664 (49.8%)	19.1% (15.9-22.2)	<0.001
Age >75y	909 (21.8%)	2371 (19.1%)	61.7% (58.6-64.5)	<0.001	1729 (18.4%)	2490 (19.7%)	30.6% (26.2-34.7)	<0.001	1650 (18.8%)	2226 (18.9%)	25.9% (21.0-30.4)	<0.001	1747 (20.1%)	2358 (20.7%)	25.9% (21.2-30.4)	<0.001
Age 65-75y	594 (14.3%)	1611 (13.0%)	63.1% (66.4-59.5)	<0.001	1228 (13.1%)	1604 (12.7%)	23.4% (17.5-28.9)	<0.001	1196 (13.6%)	1463 (12.4%)	18.3% (11.8-24.3)	<0.001	1211 (13.9%)	1592 (14.0%)	23.9% (18.0-29.4)	<0.001
Age 50-64y	894 (21.4%)	2359 (18.9%)	62.0% (58.9-64-8)	<0.001	1846 (19.6%)	2473 (19.6%)	25.4% (20.7-29.7)	<0.001	1943 (22.1%)	2427 (20.6%)	19.9% (15.0-24.6)	<0.001	1840 (21.2%)	2309 (20.3%)	20.3% (15.3-25.0)	<0.001
Age <50y	1704 (40.9%)	6017 (48.4%)	71.7% (73.2-70.1)	<0.001	3748 (39.9%)	6046 (47.9%)	38.0% (35.4-40.5)	<0.001	4018 (45.7%)	5664 (48.1%)	29.3% (26.4-32.1)	<0.001	3900 (44.9%)	5101 (44.9%)	23.5% (20.3-26.7%)	<0.001
Pediatric (<14y)	73 (1.8%)	691 (5.6%)	89.4% (86.6-91.7)	<0.001	253 (2.7%)	596 (4.7%)	57.6% (50.8-63.4)	<0.001	238 (2.7%)	396 (3.4%)	39.9% (29.4-48.8)	<0.001	195 (2.2%)	352 (3.1%)	44.6% (24.0-53.5)	<0.001
**Time of ED arrival**																
00-08	650 (15.6%)	1571 (12.6%)	58.6% (55.5-63.0)	<0.001	1361 (14.5%)	1746 (13.8%)	22.1% (16.3-27.4)	<0.001	1183 (13.4%)	1694 (14.4%)	33.1% (24.7-35.1)	<0.001	1325 (15.2%)	1689 (14.9%)	21.6% (15.7-27.0)	<0.001
08-16	2010 (48.2%)	6647 (53.5%)	69.8% (68.4-71.4)	<0.001	4719 (50.2%)	6380 (50.6%)	26.0% (23.2-28.8)	<0.001	4503 (51.2%)	5908 (50.2%)	23.8% (20.8-26.7)	<0.001	4429 (50.9%)	5663 (49.8%)	21.8% (18.5-25.0)	<0.001
16-24	1508 (36.2%)	4202 (33.8%)	64.1% (62.3-66.5)	<0.001	3324 (35.3%)	4483 (35.6%)	25.9% (22.5-29.1)	<0.001	3110 (35.4%)	4166 (35.4%)	25.3% (21.8-28.7)	<0.001	2939 (33.8%)	4013 (35.3%)	26.8% (23.2-30.2)	<0.001

cum. No. = cumulative number; %ch. = percent change.Percent values in parentheses are relative to total ED visits in each period.

Appendix Table 4. ED visit count classified by triage code and main symptom in the selected periods, with corresponding % change.

	31st Mar-13th Apr (peak period)				16th Jun-29th Jun (early post-peak period)				14th Jul-27th Jul (mid post-peak period)				18th Aug-31st Aug (late post-peak period)			
	2020	2019			2020	2019			2020	2019			2020	2019		
	cum. No.	cum. No.	%ch.	*P*	cum. No.	cum. No.	%ch.	*P*	cum. No.	cum. No.	%ch.	*P*	cum. No.	cum. No.	%ch.	*P*
**Triage codes**																
Red	165 (4.0%)	145 (1.2%)	-13.8% (-42.2-9.0)	0.256	150 (1.6%)	149 (1.2%)	-0.7% (-24.7-20.0)	0.954	147 (1.7%)	127 (1.1%)	-15.7% (-46.8-8.7)	0.227	114 (1.3%)	129 (1.1%)	11.6% (-13.7-31.3)	0.336
Yellow	657 (15.8%)	1466 (11.8%)	55.2% (50.9-59.1)	<0.001	1226 (13.0%)	1419 (11.3%)	13.6% (6.7-20.0)	<0.001	1050 (11.9%)	1302 (11.1%)	19.4% (12.5-25.7)	<0.001	1067 (12.3%)	1324 (11.6%)	19.4% (12.6-25.7)	<0.001
Green	2937 (70.5)	8200 (66.0%)	64.2% (62.6-65.7%)	<0.001	6569 (69.9%)	8279 (65.7%)	20.7% (18.0-23.2)	<0.001	5941 (67.5%)	7844 (66.7%)	24.3% (21.7-26.8)	<0.001	5728 (65.9%)	7180 (63.2%)	20.2% (17.4-23.0)	<0.001
White	409 (9.8%)	2609 (21.0%)	84.3% (82.6-85.9)	<0.001	1459 (15.5%)	2762 (21.9%)	47.2% (43.7-50.4)	<0.001	1658 (18.8%)	2495 (21.2%)	33.5% (29.3-37.5)	<0.001	1784 (20.5%)	2732 (24.0%)	34.7% (30.7-38.5)	<0.001
Urgent (red + yellow)	822 (19.7%)	1611 (13.0%)	49.0% (44.5-53.1)	<0.001	1376 (14.6%)	1568 (12.4%)	12.2% (5.7-18.4)	<0.001	1197 (13.6%)	1429 (12.1%)	16.2% (9.5-22.4)	<0.001	1181 (13.6%)	1453 (12.8%)	18.7% (12.2-24.7)	<0.001
Deferrable (white + green)	3346 (80.3%)	10809 (87.0%)	69.0% (67.8-70.2)	<0.001	8028 (85.4%)	11041 (87.6%)	27.3% (25.2-29.3)	<0.001	7599 (86.4%)	10339 (87.9%)	26.5% (24.3-28.6)	<0.001	7512 (86.4%)	9912 (87.2%)	24.2% (21.9-26.5)	<0.001
**Main symptom identified at triage**																
Dyspnea	550 (13.2%)	638 (5.1%)	13.8% (3.4-23.1)	0.011	422 (4.5%)	526 (4.2%)	19.8% (8.8-29.4)	0.001	355 (4.0%)	422 (3.6%)	15.9% (3.1-27.0)	0.016	414 (4.8%)	486 (4.3%)	14.8% (2.9-25.3)	0.017
Chest pain	195 (4.7%)	541 (4.4%)	64.0% (57.5-69.4%)	<0.001	360 (3.8%)	476 (3.8%)	24.4% (13.3-34.0)	<0.001	304 (3.5%)	452 (3.8%)	32.7% (22.2-41.8)	<0.001	278 (3.2%)	469 (4.1%)	40.7% (31.2-48.9)	<0.001
Abdominal pain	243 (5.8%)	1152 (9.3%)	78.9% (75.8-81.6)	<0.001	692 (7.4%)	1068 (8.5%)	35.2% (28.7-41.1)	<0.001	789 (9.0%)	1115 (9.5%)	29.2% (22.5-35.4)	<0.001	763 (8.8%)	1082 (9.5%)	29.5% (22.6-35.7)	<0.001
Psychiatric symptom	77 (1.8%)	180 (1.4%)	57.2% (44.1-67.2)	<0.001	152 (1.6%)	204 (1.6%)	26.0% (8.1-39.6)	0.006	167 (1.9%)	291 (2.5%)	42.6% (30.6-52.6)	<0.001	126 (1.4%)	176 (1.5%)	28.4% (10.0-43.0)	0.004

cum. No. = cumulative number; %ch. = percent change. Percent values in parentheses are relative to total ED visits in each period.

Appendix Table 5. ED visit count classified by final diagnosis in the selected periods, with corresponding % change compared to 2019.

	31st Mar-13th Apr (peak period)				16th Jun-29th Jun (early post-peak period)				14th Jul-27th Jul (mid post-peak period)				18th Aug-31st Aug (late post-peak period)			
	2020	2019			2020	2019			2020	2019			2020	2019		
	cum. No.	cum. No.	%ch.	*P*	cum. No.	cum. No.	%ch.	*P*	cum. No.	cum. No.	%ch.	*P*	cum. No.	cum. No.	%ch.	*P*
**ED final diagnosis**																
Infectious diseases	209 (5.0%)	287 (2.3%)	27.2% (13.0-39.1)	<0.001	232 (2.5%)	385 (3.1%)	39.7% (29.1-48.8)	<0.001	249 (2.8%)	362 (3.1%)	31.2% (19.2-41.5)	<0.001	276 (3.2%)	399 (3.5%)	30.8% (19.4-40.7)	<0.001
Oncological diseases	24 (0.6%)	59 (0.5%)	59.3% (34.6-74.7)	<0.001	64 (0.7%)	73 (0.6%)	12.3% (-22.6-37.3)	0.442	70 (0.8%)	78 (0.7%)	10.3% (-23.9-35.0)	0.511	67 (0.8%)	76 (0.7%)	11.8% (-22.4-36.5)	0.452
Metabolic and Endocrine diseases	39 (0.9%)	90 (0.7%)	62.7% (43.6-75.3)	<0.001	89 (0.9%)	115 (0.9%)	22.6% (-2.1-41.3)	0.069	102 (1.2%)	107 (0.9%)	4.7% (-25.0-27.3)	0.729	80 (0.9%)	96 (0.8%)	16.7% (-12.1-38.1)	0.228
Hematologic diseases	31 (0.7%)	83 (0.7%)	62.7 (56.9-67.7)	<0.001	78 (0.8%)	78 (0.6%)	0.0% (-36.9-26.9)	1.0	69 (0.8%)	84 (0.7%)	17.9% (-12.9-40.3)	0.226	48 (0.6%)	67 (0.6%)	28.4% (-3.8-50.5)	0.078
Psychiatric diseases	256 (6.1%)	686 (5.5%)	62.7% (56.9-66.2)	<0.001	633 (6.7%)	830 (6.6%)	23.7% (15.4-31.2)	<0.001	625 (7.1%)	793 (6.7%)	21.2% (12.5-29.0)	<0.001	669 (7.7%)	749 (6.6%)	10.7% (0.9-19.5)	0.034
Neurological diseases	205 (4.9%)	1065 (8.6%)	80.8% (77.6-83.4)	<0.001	684 (7.3%)	976 (7.7%)	29.9% (22.7-36.4)	<0.001	685 (7.8%)	929 (7.9%)	26.3% (18.6-31.2)	<0.001	729 (8.4%)	979 (8.6%)	25.5% (18.0-32.3)	<0.001
Cardiovascular diseases	407 (9.8%)	1075 (8.7%)	62.1% (57.6-66.2)	<0.001	844 (9.0%)	915 (7.3%)	7.8% (-1.3-16.0)	0.091	785 (8.9%)	926 (7.9%)	15.2% (6.8-22.9)	0.001	760 (8.7%)	903 (7.9%)	15.8% (7.3-23.6)	<0.001
Respiratory diseases	509 (12.2%)	788 (6.3%)	35.4% (27.8-42.2)	<0.001	343 (3.6%)	654 (5.2%)	47.6% (40.2-54.0)	<0.001	277 (3.1%)	465 (4.0%)	40.4% (30.9-48.7)	<0.001	369 (4.2%)	482 (4.2%)	23.4% (12.3-33.2)	<0.001
Gastrointestinal diseases	383 (9.2%)	1354 (10.9%)	71.7% (68.3-74.7)	<0.001	963 (10.2%)	1320 (10.5%)	27.0% (20.7-32.9)	<0.001	944 (10.7%)	1301 (11.1%)	27.4% (21.1-33.3)	<0.001	1027 (11.8%)	1346 (11.8%)	23.7% (17.2-29.7)	<0.001
Urological diseases	219 (5.3%)	682 (5.5%)	67.9% (62.6-72.4)	<0.001	544 (5.8%)	769 (6.1%)	29.3% (21.0-36.6)	<0.001	579 (6.6%)	749 (6.4%)	22.7% (13.8-30.6)	<0.001	581 (6.7%)	698 (6.1%)	16.8% (7.1-25.4)	0.001
Ob/gyn diseases	96 (2.3%)	210 (1.7%)	54.3% (41.8-64.1)	<0.001	136 (1.4%)	222 (1.8%)	38.7% (24.2-50.5)	<0.001	172 (2.0%)	212 (1.8%)	18.9% (0.8-33.6)	0.042	156 (1.8%)	208 (1.8%)	25.0% (7.7-39.1)	0.007
Trauma and burns	416 (10%)	2144 (17.3%)	80.6% (78.4-82.5)	<0.001	1627 (17.3%)	2294 (18.2%)	29.1% (24.4-33.4)	<0.001	1766 (20.1%)	2118 (18.0%)	16.6% (11.2-21.7)	<0.001	1552 (17.9%)	1752 (15.4%)	11.4% (5.2-17.3)	0.001

Syncope	29 (0.7%)	118 (1.0%)	75.4% (63.1-83.6)	<0.001	87 (0.9%)	102 (0.8%)	14.7% (-13.5-35.9)	0.276	85 (1.0%)	119 (1.0%)	28.6% (5.6-45.9)	0.018	74 (0.9%)	98 (0.9%)	24.5% (-2.1-44.2)	0.068
Unspecific chest pain	150 (3.6%)	364 (2.9%)	58.8% (50.2-65.9)	<0.001	252 (2.7%)	294 (2.3%)	14.3% (-1.4-27.6)	0.073	214 (2.4%)	300 (2.5%)	28.7% (15.0-40.1)	<0.001	205 (2.4%)	309 (2.7%)	33.7% (20.8-44.4)	<0.001

cum. No. = cumulative number; %ch. = percent change. Percent values in parentheses are relative to total ED visits in each period.

Appendix Table 6. ED outcome (admission/discharge) and admissions for non-COVID-19 diagnoses in the selected periods, with corresponding % change compared to 2019.

	31st Mar-13th Apr (peak period)				16th Jun-29th Jun (early post-peak period)				14th Jul-27th Jul (mid post-peak period)				18th Aug-31st Aug (late post-peak period)			
	2020	2019			2020	2019			2020	2019			2020	2019		
	cum. No.	cum. No.	%ch.	*P*	cum. No.	cum. No.	%ch.	*P*	cum. No.	cum. No.	%ch.	*P*	cum. No.	cum. No.	%ch.	*P*
**ED disposal**																
Hospital admission	823 (19.7%)	1360 (11.0%)	39.5% (28.6-67.7)	<0.001	1174 (12.5%)	1346 (10.7%)	12.8% (5.7-19.3)	0.001	1229 (14.0%)	1311 (11.1%)	6.3% (-1.3-13.3)	0.104	1175 (13.5%)	1338 (11.8%)	12.2% (5.0-18.8)	0.001
Home discharge	2811 (67.4%)	10084 (81.2%)	72.1% (70.9-73.3%)	<0.001	6887 (73.2%)	10258 (81.4%)	32.9% (30.8-34.9)	<0.001	7038 (80.0%)	9597 (81.6%)	26.7% (24.4-28.9)	<0.001	6723 (77.3%)	8897 (78.3%)	24.4% (22.0-26.8)	<0.001
**Hospital admission by disease group**																
Infectious diseases	36 (4.4%)	75 (5.5%)	52.0% (28.6-67.7)	<0.001	86 (7.3%)	112 (8.3%)	23.2% (-1.7-42.0)	0.065	94 (7.6%)	106 (8.1%)	11.3% (-17.1-32.8)	0.396	95 (8.1%)	100 (7.5%)	5.0% (-25.8-28.3)	0.720
Oncological diseases	15 (1.8%)	34 (2.5%)	55.9% (19.0-76.0)	0.008	38 (3.2%)	31 (2.3%)	-22.6% (-97.0-23.7)	0.400	42 (3.4%)	38 (2.9%)	-10.5% (-71.4-28.7)	0.655	31 (2.6%)	31 (2.3%)	0.0% (-64.5-39.2)	1.0
Metabolic and Endocrine diseases	18 (2.2%)	32 (2.4%)	43.7% (-0.2-68.4)	0.051	31 (2.6%)	34 (2.5%)	8.8% (-48.3-44.0)	0.710	39 (3.2%)	36 (2.7%)	-8.3% (-71.4-28.7)	0.729	34 (2.9%)	31 (2.4%)	-6.3% (-72.2-34.4)	0.806
Hematologic diseases	14 (1.7%)	25 (1.8%)	44.0% (-7.7-70.9)	0.082	24 (2.0%)	25 (1.9%)	4.0% (-68.1-45.2)	0.886	19 (1.5%)	25 (1.9%)	24.0% (-38.0-58.1)	0.397	18 (1.5%)	23 (1.7%)	21.7% (-45.0-57.8)	0.436
Psychiatric diseases	24 (2.9%)	72 (5.3%)	66.7% (47.1-79.0)	<0.001	60 (5.1%)	92 (6.8%)	34.8% (9.7-52.9)	0.010	59 (4.8%)	81 (6.2%)	27.2% (-1.9-47.9)	0.064	60 (5.1%)	90 (6.7%)	33.3% (7.6-51.9)	0.015
Neurological diseases	18 (2.2%)	36 (2.6%)	50.0% (12.0-71.6)	0.016	30 (2.6%)	45 (3.3%)	33.3% (-5.8-58.0)	0.085	50 (4.1%)	36 (2.7%)	-38.9% (-113.2-9.5)	0.133	35 (3.0%)	38 (2.8%)	7.9% (-45.8-41.8)	0.726
Cardiovascular diseases	184 (22.4%)	326 (24.0%)	43.6% (32.4-52.9)	<0.001	318 (27.1%)	272 (20.2%)	-16.9% (-37.5-0.6)	0.059	302 (24.6%)	284 (21.7%)	-6.3% (-25.0-9.6)	0.457	269 (22.9%)	278 (20.8%)	3.2% (-14.4-18.2)	0.700
Respiratory diseases	200 (24.3%)	216 (15.9%)	7.4% (-12.2-23-6)	0.433	118 (10.1%)	190 (14.1%)	37.9% (21.9-50.6)	<0.001	115 (9.4%)	140 (10.7%)	17.9% (-5.1-35.8)	0.118	114 (9.7%)	131 (9.8%)	13.0% (-11.9-32.3)	0.278
Gastrointestinal diseases	86 (10.4%)	165 (12.1%)	47.9% (32.4-59.8)	<0.001	143 (12.2%)	160 (11.9%)	10.6% (-12.0-28.7)	0.329	161 (13.1%)	194 (14.8%)	17.0% (-2.3-32.7)	0.080	161 (13.7%)	197 (14.7%)	18.3% (-0.6-33.6)	0.058

Urological diseases	37 (4.5%)	78 (5.7%)	52.6% (29.9-67.9)	<0.001	67 (5.7%)	86 (6.4%)	22.1% (-7.2-43.4)	0.126	75 (6.1%)	88 (6.7%)	14.8% (-16.0-37.4)	0.309	92 (7.8%)	92 (6.9%)	0.0% (-33.5-25.1)	1.0
Ob/gyn diseases	29 (3.5%)	52 (3.8%)	44.2% (12.2-64.6)	0.012	21 (1.8%)	39 (2.9%)	46.2% (8.5-68.3)	0.022	34 (2.8%)	46 (3.5%)	26.1% (-15.1-52.6)	0.181	33 (2.8%)	56 (4.2%)	41.1% (9.4-61.7)	0.016
Trauma and Burns	48 (5.8%)	61 (4.5%)	21.3% (-14.9-46.1)	0.214	66 (5.6%)	89 (6.6%)	25.8% (-2.0-46.1)	0.066	78 (6.3%)	72 (5.5%)	-8.3% (-49.2-21.4)	0.624	299 (25.4%)	337 (25.2%)	11.3% (-3.7-24.1)	0.132
Pediatric admissions	3 (0.4%)	34 (2.5%)	91.2% (71.3-97.3)	<0.001	6 (0.5%)	24 (1.8%)	75.0% (38.8-89.8)	0.002	10 (0.8%)	18 (1.4%)	44.4% (-20.3-74.4)	0.136	11 (0.9%)	33 (2.5%)	66.7% (34.0-83.2)	0.002
Syncope	2 (0.2%)	12 (0.9%)	83.3% (25.5-96.3)	0.019	13 (1.1%)	2 (0.9%)	-8.3% (-137.4-50.6)	0.842	14 (1.1%)	18 (1.4%)	22.2% (-56.4-61.3)	0.481	9 (0.8%)	16 (1.2%)	43.7% (-27.3-75.1)	0.167
Unspecific chest pain	6 (0.6%)	8 (0.6%)	25.0% (-116.2-74.0)	0.594	10 (0.9%)	8 (0.6%)	-25.0% (-216.7-50.7)	0.638	17 (1.4%)	8 (0.6%)	-112.5% (-392.4-8.3)	0.079	6 (0.5%)	11 (0.8%)	45.5% (-47.5-79.8)	0.232

cum. No. = cumulative number; %ch. = percent change. % values of hospital admissions and home discharges are relative to total ED visits in each period. % values of hospital admissions by disease group are relative to total hospital admissions in each period.

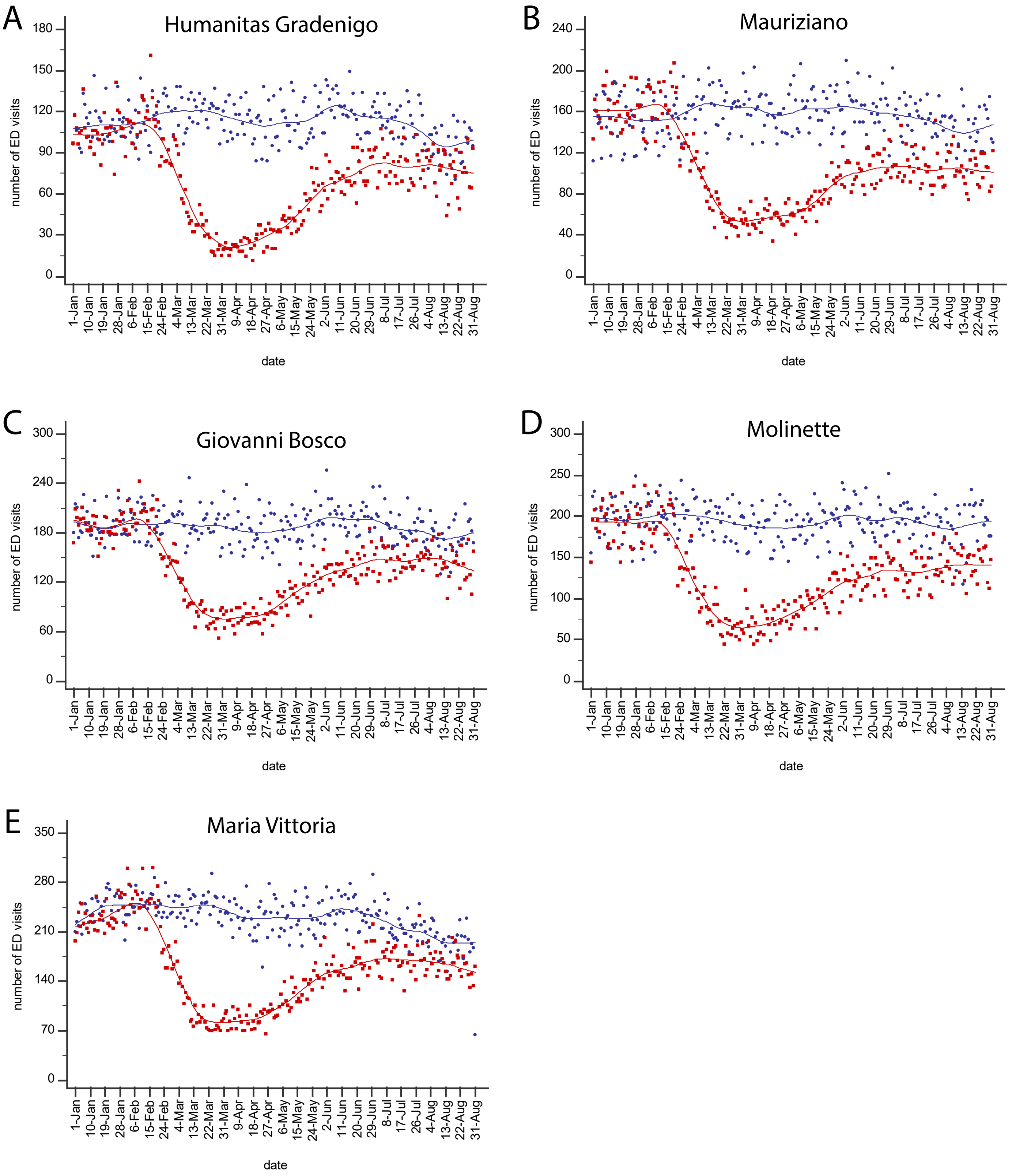

Appendix figure 1. Number of ED visits in each participant center (A to E). Red dots/line represent data in 2020, blue dots/line represent data in 2019.
